# Commentary: The reliability and quality of short videos as health information of guidance for lymphedema: a cross-sectional study

**DOI:** 10.3389/fpubh.2025.1605011

**Published:** 2025-06-30

**Authors:** Henrique Jose Pereira de Godoy, Jose Maria Pereira de Godoy

**Affiliations:** ^1^Vascular Surgery Discipline of the Medicine School in São do José do Rio Preto (FAMERP), São Paulo, Brazil; ^2^Cardiology and Cardiovascular Surgery Department of the Medicine School in São José do Rio Preto (FAMERP), Head Vascular Surgery Discipline-FAMERP and Research CNPq (National Council for Research and Development), São José do Rio Preto, Brazil

**Keywords:** lymphedema, short videos, health, information, guidance

## Introduction

The interesting article entitled “The reliability and quality of short videos as health information of guidance for lymphedema: a cross-sectional study” (1), published in this journal reports on an extremely important subject related to treatment within health care, specifically in the areas of surgery and physiotherapy. Medical treatment must be based on scientific evidence, otherwise it cannot be considered a therapeutic option but rather an approach using guesswork, which is scientifically condemned.

## Subsections

It is common on social media for people without sufficient knowledge of the scientific basis for treating lymphedema to ‘invent' a form of treatment and publish it in social networking sites. Laypeople, believing that it is a reliable form of treatment, begin to use it. Even non-specialist professionals have created methods without the slightest scientific basis or evidence and published them on social media platforms. This is the start of the destruction of the scientific foundations of medicine resulting in a loss of credibility.

## Discussion

As a vascular surgeon and lymphology specialist with over 35 years of experience and extensive publications in the field, my team and I have observed that many online videos related to lymphology contain misinformation that could be detrimental to patients. This aligns with our daily clinical practice, where we see firsthand the negative consequences of inexperienced practitioners. Therefore, it is crucial to ensure the quality and accuracy of such videos.

When we analyze these videos, we need to have scientific knowledge of the content, so it is not the number of publications that is important, but the quality of the material. We must also examine how treatment techniques for these patients have advanced over the past century (2–5), evaluating the resulting improvements in patient outcomes and the innovative scientific approaches driving these changes. Because of improvements in scientific research, new techniques, new concepts and new materials are far superior in terms of results compared to the last century.

Specifically in lymphedema, we do not have favorable or contraindicating studies, in some other areas with other types of social platforms such as YouTube, Tick-Tok and in general the recommendation the videos and platforms should strengthen the management and professional supervision of related videos to accurately disseminate relevant knowledge to patients (6, 7). Just as we previously reported, the quality of the videos are role in health information dissemination expands, ensuring but must guarantee quality scientific and up-to-date content to better meet patients' needs for information.

In short, social media can do more harm to medicine than it contributes to the treatment of patients, and so warnings about these risks are of fundamental importance.
